# Regulation of Bottromycin Biosynthesis Involves an Internal Transcriptional Start Site and a Cluster-Situated Modulator

**DOI:** 10.3389/fmicb.2020.00495

**Published:** 2020-03-26

**Authors:** Natalia M. Vior, Eva Cea-Torrescassana, Tom H. Eyles, Govind Chandra, Andrew W. Truman

**Affiliations:** Department of Molecular Microbiology, John Innes Centre, Norwich, United Kingdom

**Keywords:** *Streptomyces*, bottromycin, RiPP, antibiotic, regulation, transcription, RNA-seq, natural product

## Abstract

Bottromycin is a ribosomally synthesized and post-translationally modified peptide (RiPP) produced by several streptomycetes, including the plant pathogen *Streptomyces scabies*. There is significant interest in this molecule as it possesses strong antibacterial activity against clinically relevant multidrug resistant pathogens and is structurally distinct from all other antibiotics. However, studies into its efficacy are hampered by poor yields. An understanding of how bottromycin biosynthesis is regulated could aid the development of strategies to increase titres. Here, we use 5′-tag-RNA-seq to identify the transcriptional organization of the gene cluster, which includes an internal transcriptional start site that precedes *btmD*, the gene that encodes the bottromycin precursor peptide. We show that the gene cluster does not encode a master regulator that controls pathway expression and instead encodes a regulatory gene, *btmL*, which functions as a modulator that specifically affects the expression of *btmD* but not genes up- or downstream of *btmD*. In order to identify non-cluster associated proteins involved in regulation, proteins were identified that bind to the main promoter of the pathway, which precedes *btmC*. This study provides insights into how this deceptively complex pathway is regulated in the absence of a pathway specific master regulator, and how it might coordinate with the central metabolism of the cell.

## Introduction

**FIGURE 1:**
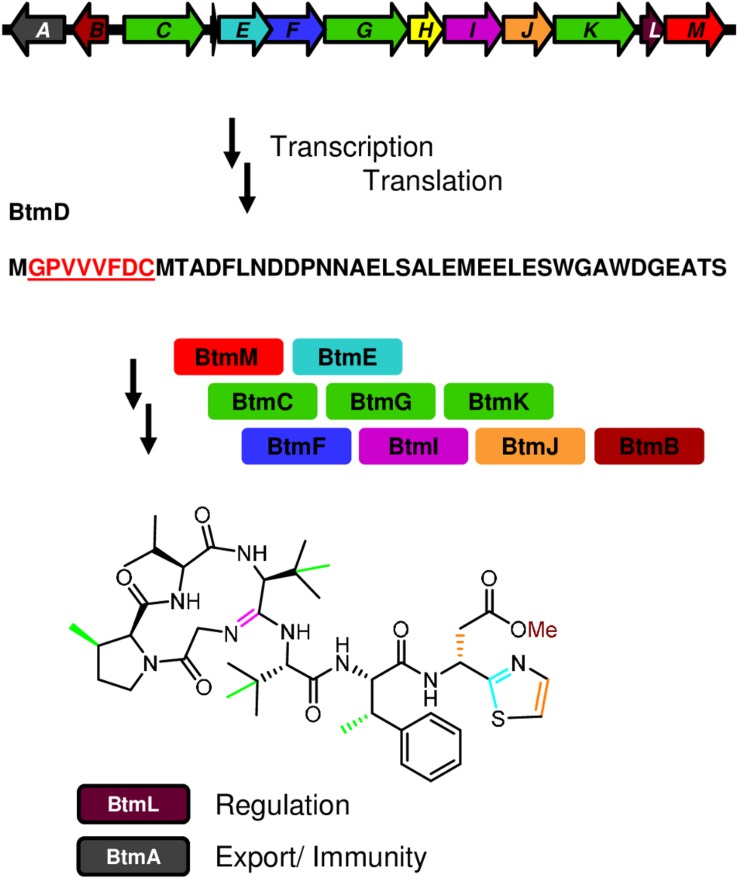
Biosynthesis of bottromycin. The diagram shows the genetic organization of the bottromycin (*btm*) cluster and summarizes its biosynthetic pathway, from the transcription and translation of the precursor peptide gene *btmD*, to the posttranslational modifications leading to the final compound.

Infectious diseases are responsible for one-fifth of deaths worldwide, and antimicrobial resistance is becoming an increasingly serious threat to global public health, which makes the development of new antibiotics a pressing need (Laxminarayan et al., 2016; Martens and Demain, 2017). The effective treatment of multidrug resistant bacteria requires the identification of compounds with either unusual chemical scaffolds or novel molecular targets, capable of overcoming multiple and widespread resistance mechanisms (Laxminarayan, 2014; Newman and Cragg, 2016; Martens and Demain, 2017). One antimicrobial compound of great interest is bottromycin, a peptide antibiotic with promising activity against multi-drug resistant Gram-positive bacterial pathogens (Shimamura et al., 2009; Tacconelli and Magrini, 2017). Bottromycin was first isolated in 1957 from cultures of *Streptomyces bottropensis* (Waisvisz et al., 1957), and then more recently as a product of the plant pathogen *Streptomyces scabies* (Crone et al., 2012) and other *Streptomyces* species (Gomez-Escribano et al., 2012; Hou et al., 2012; Huo et al., 2012). Bottromycin is a ribosomally synthesized and post-translationally modified peptide (RiPP). In its biosynthetic pathway, a precursor peptide (BtmD) undergoes a complex and unprecedented series of modifications catalyzed by enzymes encoded in the bottromycin (*btm*) gene cluster (Figure 1) (Crone et al., 2012, 2016; Gomez-Escribano et al., 2012; Huo et al., 2012; Franz et al., 2017; Schwalen et al., 2017; Sikandar et al., 2019).

Bottromycin is a structurally novel compound that selectively blocks the binding of aminoacyl t-RNAs to the A-site in the 50S subunit of bacterial ribosomes, ultimately leading to protein synthesis inhibition (Otaka and Kaji, 1976, 1981, 1983). This makes bottromycin an interesting lead for development, as it attacks a molecular target that is currently unexploited in the clinic. However, bottromycin is produced in low yields, where titres in wild type producers are below 1 mg/L (Huo et al., 2012). These low production levels complicate both drug development and further biosynthetic studies of these molecules (Huo et al., 2012; Crone et al., 2016). An understanding of how bottromycin biosynthesis is regulated may enable the rational improvement of yield by modifying pathway regulation, especially as the pathway is inefficient in laboratory conditions (Crone et al., 2016; Eyles et al., 2018).

It is very well documented that altering the regulatory networks that control secondary metabolite biosynthesis often leads to significant yield increases, either by overexpressing positive regulators, inactivating repressors, or altering higher levels of regulation in the producer strain (Makitrynskyy et al., 2013; Vior et al., 2014; Yoo et al., 2015; Huang et al., 2016). RiPP biosynthesis, especially in the case of lantibiotics, is often regulated by classic two-component systems or orphan response regulators (Bierbaum and Sahl, 2009; Cooper et al., 2010), but this is not a mechanism common to all RiPP pathways. Systems involving sigma-anti-sigma factor pairs, SARP, LuxR, or TetR regulators have also been documented (Izawa et al., 2013; Flinspach et al., 2014; Zhang et al., 2014; Fernández-Martínez et al., 2015), and more diverse regulatory systems are likely to be found in the future, especially because many RiPP gene clusters do not encode any obvious pathway specific regulators (Bartholomae et al., 2017). The *btm* gene cluster in *S. scabies* encodes one potential regulatory protein, BtmL (Figure 1) (Crone et al., 2012). This protein is conserved across all characterized bottromycin gene clusters (Crone et al., 2012; Gomez-Escribano et al., 2012; Huo et al., 2012), but nothing is known about how this putative regulator controls bottromycin biosynthesis, nor whether additional regulators have critical roles in *btm* cluster regulation.

In this work, we apply transcriptomic, proteomic and metabolomic techniques in combination with qRT-PCR and reporter activity experiments in order to obtain key details on the regulation of bottromycin biosynthesis. We show that BtmL is not a master regulator of biosynthesis and instead specifically enhances expression levels of the precursor peptide gene *btmD*. This occurs in conjunction with a transcriptional start site for *btmD* that is internal to the preceding gene in the cluster. We show that this pathway is surprisingly complex and provide evidence into how it is regulated in the absence of a pathway specific master regulator.

## Results

### BtmL Specifically Modulates the Expression of the Precursor Peptide Gene *btmD* and Is Independent of Cobalt Levels in the Medium

The *btm* gene cluster in *S. scabies* encodes a single putative regulator, BtmL, a 20.5 kDa protein that contains a *C*-terminal domain of unknown function (DUF2087 or PF09860), which has been associated with putative transcriptional regulators and is proposed to bind nucleic acids (Rigden, 2011). To date (July 2019), over 6,500 DUF2087-containing proteins have been sequenced and deposited in Genbank, of which more than 2,000 have additional DNA-binding domains. *In silico* analysis of BtmL using both Phyre2 (Crone et al., 2012; Kelley et al., 2015) and I-TASSER (Yang et al., 2015) predicted that the *N*-terminus of the protein has structural homology with SmtB-ArsR-like repressors, and therefore would feature a winged helix-turn-helix (wHTH) domain characteristic of this family of transcriptional regulators (Busenlehner et al., 2003; Osman and Cavet, 2010; Chakravorty and Merz, 2014). However, no canonical wHTH domain was detected by sequence analysis and the protein appears to lack the conserved residues characteristically involved in ArsR-family metal binding (Osman and Cavet, 2010; Crone et al., 2012). Despite this, previous empirical evidence showed that addition of cobalt(II) to the production medium increased bottromycin yields (Crone et al., 2012), inferring that the ArsR-like structure of BtmL may control cluster expression via a metal-binding mechanism. This hypothesis was especially compelling given that several genes in the *btm* cluster encode class B radical SAM methyltransferases, which are cobalamin-dependent enzymes (Bauerle et al., 2015).

**FIGURE 2:**
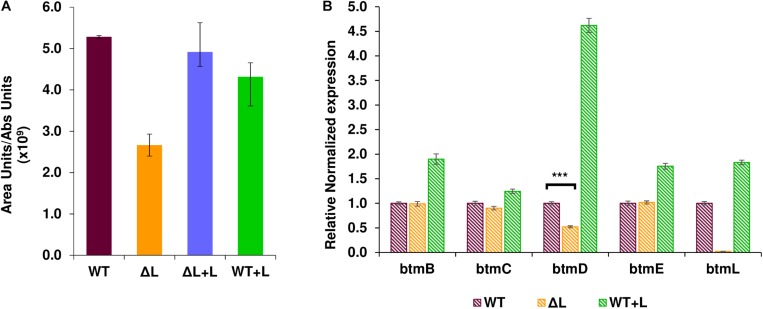
Effect of *btmL* on bottromycin production and *btm* gene transcription. **(A)** LC-MS analysis of bottromycin production in *S. scabies* WT, Δ*btmL* (ΔL), ΔL complemented with a copy of *btmL* (ΔL + L) and the WT strain overexpressing *btmL* (WT + L). Error bars represent the standard deviation of the average production values in biological triplicates, which are normalized by culture growth. **(B)** qRT-PCR analysis of the transcription of representative *btm* genes in the WT (purple bars), Δ*btmL* (yellow bars) and WT + L (green bars) at 72 h of growth in BPM. Expression values are relative to the expression of the target gene in the WT, which was set at 1. Transcription of *hrdB* was used to normalize the expression levels across samples. Error bars represent standard error of the mean from triplicate experiments, and the asterisk represents a statistically significant difference in *btmD* expression between wt and the mutant Δ*btmL* (*p*-value < 0.001).

To investigate the role of BtmL in bottromycin biosynthesis, a mutant *S. scabies* strain carrying an in-frame deletion of *btmL* was generated (Crone et al., 2012) and its ability to produce bottromycin was assessed using liquid chromatography - mass spectrometry (LC-MS). Inactivation of *btmL* did not abolish bottromycin biosynthesis, but we could observe a moderate and consistent decrease in production levels to approximately 40% of wild type (WT) levels (Figure 2A). This result suggests that *btmL* acts as a positive modulator of bottromycin biosynthesis, but is not the master activator of the pathway. WT levels of bottromycin production were restored in Δ*btmL* upon *in trans* complementation with a copy of *btmL* under the control of the constitutive promoter *ermE*^∗^p (Bibb et al., 1985), confirming that the phenotype was due to the deletion of *btmL*. Surprisingly, when that same *btmL* expression construct was introduced in the WT strain (generating strain WT + L), there was no increase in bottromycin levels (Figure 2A).

To evaluate quantitatively whether *btmL* could be acting as a transcriptional regulator, qRT-PCR experiments were carried out with RNA samples from both the WT and Δ*btmL* strains (Figure 2B). The *btm* gene cluster is organized in two divergent groups of genes: *btmA* and *btmB* on one strand and *btmC*-*btmM* on the other strand (Figure 1). We therefore measured the expression of *btmB* from the smaller block, and *btmC*, *btmD* (the gene encoding the precursor peptide), *btmE* and *btmL* from the rest of the cluster. Expression values of these genes were normalized using the expression of *hrdB*, which encodes the principal sigma factor of RNA polymerase. A time course experiment determined that the optimal time to measure *btm* gene expression was at 72 h post-inoculation of bottromycin production medium (Supplementary Figure S1). At this time point, it could be observed that deletion of *btmL* has no significant effect on most of the genes tested, with one notable exception: *btmD*, the gene encoding the bottromycin precursor peptide. Transcription of *btmD* is reduced to approximately 40% of WT levels, which correlates with the reduction in bottromycin production (Figure 2B). Furthermore, qRT-PCR analysis of the WT + L strain revealed that transcription of *btmD* is significantly increased in this strain, confirming that BtmL is a specific and positive regulator of *btmD*. Interestingly, upstream and downstream genes (*btmB*, *btmC*, and *btmE*) are only slightly overexpressed or not overexpressed at all, suggesting the presence of an alternative operon encompassing only *btmD*.

**FIGURE 3:**
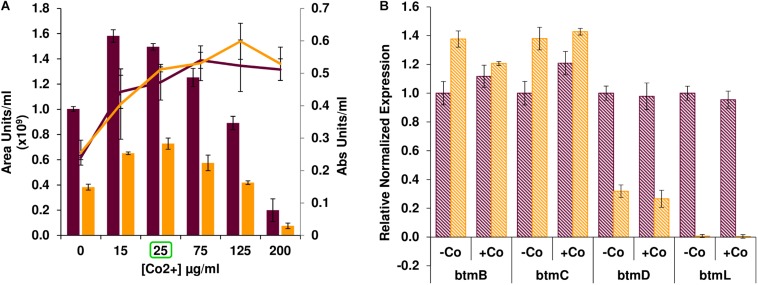
Effect of cobalt(II) on bottromycin biosynthesis. **(A)** Bottromycin production in *S. scabies* WT (purple bars) and Δ*btmL* (yellow bars) in BPM supplemented with increasing concentrations of CoCl_2_. Culture growth (calculated from a DNA quantification assay, see section “Materials and Methods”) is overlaid and represented on the secondary axis. Error bars represent the standard deviation of biological triplicate data. The concentration of cobalt used for the qRT-PCR experiment in **(B)** appears highlighted with a green square. **(B)** qRT-PCR analysis of the expression of *btm* genes in the WT (purple bars) and Δ*btmL* mutant (yellow bars) at 72 h of growth in BPM with and without added cobalt. Expression values are relative to the expression of the target gene in the WT without added cobalt, which was set at 1. Transcription of *hrdB* was used to normalize the expression levels across samples and error bars represent standard error of the mean from triplicate experiments.

After confirming that *btmL* is involved in bottromycin regulation, we assessed whether cobalt(II) levels influence pathway productivity, and whether this effect was mediated by *btmL*. Bottromycin production was measured in *S. scabies* WT and Δ*btmL* cultured in bottromycin production medium (BPM) supplemented with different concentrations of CoCl_2_ (Figure 3A). In absolute terms, bottromycin production increased upon addition of cobalt(II), reaching a maximum at 15–25 μg/mL CoCl_2_ after which production steadily decreased until almost disappearing at 200 μg/mL CoCl_2_. However, the increase in production simply corresponded to a proportional increase in mycelial growth upon addition of up to 25 μg/mL of cobalt(II) in the medium (Figure 3A). At higher cobalt(II) concentrations mycelial growth was sustained, so the drop in production was probably due to a general inhibitory effect of excess metal in the medium, a phenomenon reported for the biosynthesis of other antibiotics (Rogers and Birnbaum, 1974; Abbas and Edwards, 1990). The growth-dependent production increase was also noticeable in Δ*btmL*, which followed a similar pattern, albeit at a reduced level of production. Moreover, a comparison of qRT-PCR data for the expression levels of several pathway genes in the presence or absence of added cobalt(II) (Figure 3B) showed that these are nearly identical in either condition for both the WT and Δ*btmL* strains, discarding any specific regulatory effect of this metal. This result is in accordance with the observation that *btmL* lacks the conserved metal-binding residues characteristic of ArsR-SmtB regulators (Osman and Cavet, 2010; Crone et al., 2012).

These results ruled out cobalt(II)-dependent pathway regulation by BtmL, which is instead a cobalt-independent transcriptional modulator that selectively increases *btmD* transcript levels in order to generate high levels of the precursor peptide in a feed-forward loop. Other positive loop systems have been reported in the biosynthesis of several RiPPs like microbisporicin and planosporicin, where the autoinduction mechanism relies of the detection of small quantities of precursor peptide by a sigma-antisigma complex (Sherwood and Bibb, 2013; Fernández-Martínez et al., 2015), or cinnamycin, where production is launched after onset of immunity to the compound in the producer strain (O’Rourke et al., 2017).

### Overexpression of Exporter BtmA Has a Moderate Positive Effect on Bottromycin Production

The discordance between the increase in *btmD* expression and the lack of change in bottromycin production in the WT + L strain (Figure 2A) led us to postulate that the potential increase in available BtmD might not be effectively channeled by the pathway and exported out of the cell, leading to increased degradation of pathway intermediates and generation of shunt metabolites. Supporting this hypothesis, previous work showed that overexpression of *botT*, the major facilitator superfamily (MFS) transporter gene (Kumar et al., 2016; Quistgaard et al., 2016) in the bottromycin cluster from *Streptomyces* sp. BC16019 increased bottromycin production in a heterologous host, although it still did not reach wild type production levels (Huo et al., 2012). We therefore assessed whether overexpressing the homolog of this gene (*btmA*, 88% identity to *botT*) in the *S. scabies btm* gene cluster might increase the efficiency of the pathway and self-resistance to potentially toxic levels of the antibiotic.

**FIGURE 4:**
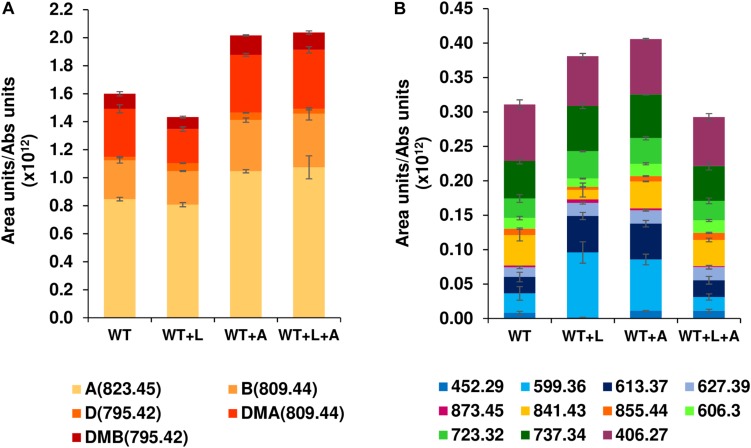
Effect of the MFS transporter gene *btmA* on bottromycin production. Comparison of WT and WT + L strains with strains overexpressing *btmA* (WT + A and WT + L + A). **(A)** Production of mature bottromycins. **(B)** Production of intermediates and shunt products of the bottromycin pathway. Legends show all the compounds quantified in the experiment, and each compound is labeled with its observed mass as described in Crone et al. (2016) and Eyles et al. (2018). Mature bottromycins are also labeled with letters (A: bottromycin A_2_, B: bottromycin B, D: bottromycin D, DMA: desmethyl bottromycin A_2_ and DMB: desmethyl bottromycin B). Error bars represent the standard deviation of biological triplicate data, and production values are normalized by culture growth.

Expression of a second copy of *btmA* under the control of the constitutive promoter *ermE*^∗^p provided a 25% increase in bottromycin production with respect to the WT strain (Figure 4A), an effect that was maintained, but not improved, when it was expressed in WT + L (generating WT + L + A). When the quantification was extended to other bottromycin related metabolites described previously (Crone et al., 2016; Eyles et al., 2018) this pattern was still observed for the total set of mature bottromycins (those including the main posttranslational modifications) (Figure 4A). However, in the case of the pathway

intermediates and shunt metabolites the trend was not present, with WT + L and WT + A producing more of them than the WT and the double overexpression strain WT + L + A (Figure 4B). This result indicates that efficiency can be increased to some extent by improving export of the molecule, but this is not the major bottleneck for bottromycin production. The main limiting factor for bottromycin production is likely to be the post-translational maturation of the precursor peptide instead, as we previously showed that the bottromycin pathway inefficiently stalls at numerous biosynthetic steps, even in the WT strain (Crone et al., 2016).

### Mapping of the Transcriptional Organization of the *btm* Cluster Identifies a Secondary Transcriptional Start Site for *btmD*

**FIGURE 5:**
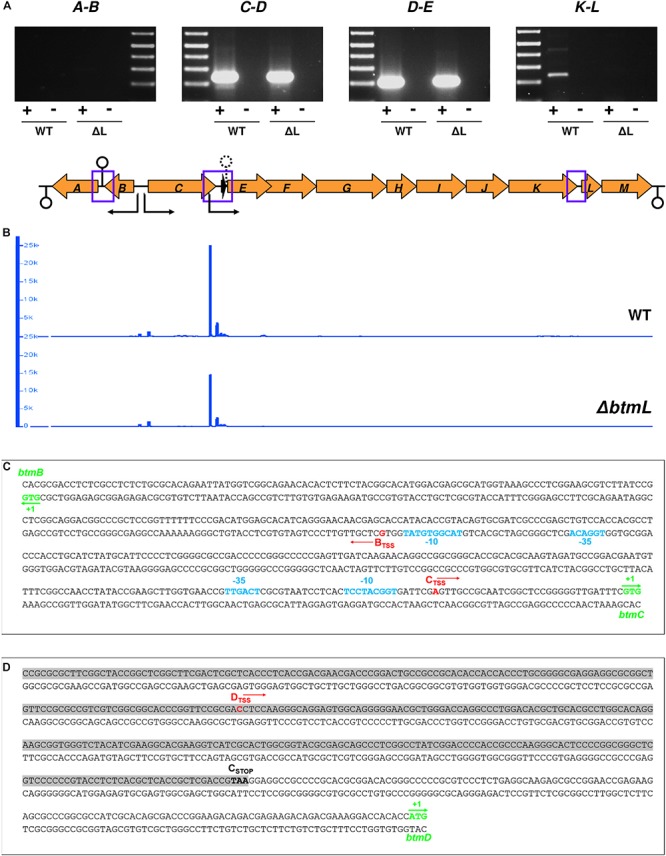
Transcriptional organization of the *btm* gene cluster. **(A)** RT-PCR cotranscription analysis of the intergenic regions with no overlapping genes in the *btm* cluster (highlighted with blue squares in the schematic representation of the *btm* gene cluster below the gel images; loop symbols in this diagram indicate the location of predicted transcription terminators -solid line- and attenuators -dashed line- in the cluster). **(B)** Transcription start site mapping of the *btm* cluster using 5′-tag-RNA-seq. The plots (generated in IGB) show the mapping of reads with TSS tags in the *btm* cluster in the WT (top) and Δ*btmL* (bottom) strains. Sharp peaks corresponding to mapped reads precede *btmB*, *btmC*, and *btmD*, along with some non-specific transcription initiation in the *btmD* region. The vertical axis in the plots represents read counts and the horizontal axis maps to the gene cluster shown in **(A)**. The direction of transcription from the identified TSSs is represented with black arrows in the cluster. **(C)** Precise mapping of TSS and potential promoter regions in the *btmB-C* intergenic region. *btmB* and *btmC* start codons are marked in green, and the TSSs identified for *btmB* and *btmC* using both 5′tag-RNA-seq and 5′-RACE are labeled in red. −10 and −35 promoter regions for *btmC* (predicted with BPROM) and *btmB* (annotated manually) are represented in light blue. **(D)** Mapping of *btmD* TSS (red) within the coding sequence of *btmC* (shadowed in gray, with its stop codon highlighted in bold).

Given that *btmD* appears to be situated within a larger operon, the specific increase in *btmD* transcription following *btmL* overexpression was unexpected and prompted us to investigate the transcriptional organization of the *btm* gene cluster. This cluster contains 13 genes, where 11 (*btmC-M*) are arranged in the same orientation and appear tightly clustered, with *btmE* to *btmJ* physically overlapping and *btmK* starting 8 bp after the *btmJ* stop codon. To assess the possibility of co-transcription of the remaining genes, RT-PCR experiments were performed on 72 h cultures of both WT and Δ*btmL* strains with specific primer pairs for the intergenic regions *btmA-B*, *btmC-D*, *btmD-E*, and *btmK-L* (Figure 5A). These showed co-transcription of all of the tested regions except *btmA-B*, indicating that genes *btmC-M* co-transcribe and can behave as a single 14.5 kb operon, whereas *btmA* and *btmB* are transcribed separately. The gene cluster was analyzed for putative terminators using WebGeSTer (Mitra et al., 2011). Consistent with the RT-PCR results, terminators were predicted spanning up to 90 bp away from the stop codon of *btmA* (Δ*G* = −19.5), between *btmA* and *btmB* (Δ*G* = −19.43) and 149 bp away from the stop codon of *btmM* (Δ*G* = −19.27) (Figure 5A).

This evidence for *btmC-M* co-transcription did not exclude the possibility of alternative operons, and the specific increase in *btmD* transcription following *btmL* overexpression hinted toward an alternative transcription start site (TSS) for *btmD* that would explain how it is differentially regulated from its surrounding genes. In order to map the TSSs for the whole cluster we employed 5′-tag-RNA-seq (also called tagRNA-seq) (Innocenti et al., 2015) that enables the identification of TSSs in an untargeted, genome-wide fashion. This powerful technique has the advantage of differentially distinguishing primary transcripts (those generated by an RNA polymerase and therefore coming from true TSSs) from processed transcripts, which arise upon degradation or RNase mediated cleavage of the original transcripts at specific processing sites (PSs). In this particular case, with an operon spanning over 10 kb, degradation or post-transcriptional modification of the resulting transcript could also account for the difference between *btmD* expression and the remainder of the *btmC-M* operon.

5′-tag-RNA-seq reads mapped to the *S. scabies* 87-22 genome clearly showed the presence of three TSSs in the *btm* cluster: one preceding *btmB*, one before *btmC*, and an extra one preceding *btmD* and within the coding region of the upstream gene, *btmC* (Figure 5B). Unexpectedly, no TSS was found before *btmA*, in contrast with what was expected from the intergenic RT-PCR result (Figure 5A). To independently confirm the existence of these TSSs, we performed 5′-RACE (Rapid amplification of cDNA ends) experiments for *btmB*_TSS_, *btmC*_TSS_, and *btmD*_TSS_, which yielded identical results to the 5′tag-RNA-seq (Figures 5C,D). *btmC*_TSS_ is in position -32 with respect to the *btmC* start codon, and a prediction of putative σ70 promoters in the *btmB-btmC* intergenic region using BPROM (Solovyev and Salamov, 2011) identified a nearly canonical −35 sequence (TTGACT) and a poorly conserved −10 region (TCCTACGGT) that are consistent with the location of the *btmC*_TSS_ (Figure 5C). It was not possible to confidently identify −10 and −35 boxes for the *btmB*_TSS_ using BPROM or other prediction tools, so potential sequences were identified manually (Figure 5C). The *btmD*_TSS_ is located 348 bp away from the start of *btmD* and 215 bp before the end of *btmC* coding sequence (Figure 5D), but we could not identify any suitable −10 or −35 regions for this TSS, either manually or with predictive tools. Unexpectedly, *btmD*_TSS_ appears both in the WT and in Δ*btmL*, albeit with lower intensity in Δ*btmL* (Figure 5B), which indicates that although *btmL* does affect the transcription of *btmD*, it is not essential for the expression of this alternative transcript.

Interestingly, the 5′tagRNA-seq results showed that at 72 h of growth there is an extremely high level of transcription of some *btm* genes, in particular *btmD*. Transcription initiation preceding this gene seems rather promiscuous with some background noise (Figure 5B). Strikingly, an analysis of the full transcriptome to assess levels of gene expression as reads per kilobase per million reads (RPKM) and transcripts per million (TPM) (Wagner et al., 2012), showed that *btmD* and *btmC* are the 4th and 9th most highly transcribed genes in the genome at this time point (Supplementary Datasheet S1). Transcription after *btmD* drops considerably, showing that even upon overexpression of *btmL*, expression of *btmE* did not increase in a comparable way to *btmD*, in accordance with the qRT-PCR results (Figure 2B). The transcriptomic data also shows that all downstream genes in the operon are transcribed at lower levels than their preceding genes. This may be explained by a transcription attenuator downstream of *btmD* that likely serves to dampen transcription of downstream genes in the cluster. Accordingly, a putative terminator sequence between *btmD* and *btmE* (Δ*G* = −21.22) is predicted by WebGeSTer (Mitra et al., 2011).

#### Genome-Wide Analysis of Transcriptional Start Sites in *S. scabies*

In RiPP biosynthesis, it appears to be beneficial to evolve mechanisms to selectively increase precursor peptide production in relation to catalytic proteins, given that precursor peptides are structural rather than catalytic. The mechanisms by which this happens are poorly understood, especially in pathways where the precursor peptide is apparently co-transcribed with upstream genes encoding catalytic proteins. The secondary transcriptional start site for *btmD* provides a previously unobserved mechanism by which this can occur. To assess whether this strategy was apparent in other RiPP gene clusters in *S. scabies*, we assessed their transcriptional organization using the 5′tagRNA-seq dataset. This showed that for all of the putative RiPP clusters identified in the *S. scabies* genome, the precursor peptide gene always has its own TSS (Supplementary Figures S2, S3). This happens even when the gene is preceded by genes predicted to encode tailoring enzymes (Supplementary Figure S2). Notably, in the case of SCAB_32021, which is tightly clustered with a preceding gene encoding a putative methyltransferase, its dedicated TSS overlaps with the end of this gene, in a similar scenario to *btmC* and *btmD* (Supplementary Figure S2). This suggests that having a dedicated TSS could be a widespread strategy for obtaining the appropriate stoichiometry of precursor peptide and tailoring enzymes in those cases where the precursor peptide gene is not at the beginning of an operon.

### Reporter Assays Show Strong Transcription From the *btmC* Promoter Yet Negligible Transcription From the Putative *btmD* Promoter

**FIGURE 6:**
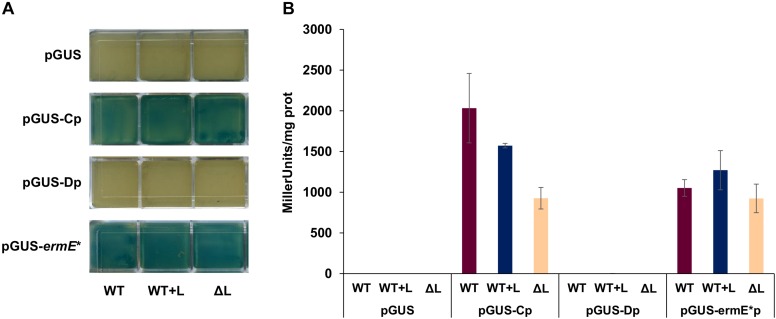
Promoter activity using β-glucuronidase (GUS) reporter assays. The putative promoter regions for *bmtC* and *btmD* were inserted before the *gusA* gene in plasmid pIJ10740 to create transcriptional fusions. The promoterless vector was used as a negative control, and pIJ10741 (containing *gusA* under the control of ermE*p) was used as a positive control. **(A)** Qualitative GUS assay in solid medium. Each of the squares represents one independent culture, with squares in the same row corresponding to strains containing the construct listed to their left, and the annotation at the bottom of each column indicating the genetic background. **(B)** Quantitative GUS assay in liquid medium, with reporter activity measured as Miller units/mg protein.

To further characterize the main promoter regions in the pathway, promoter activity was measured using the β-glucuronidase reporter gene *gusA* (Myronovskyi et al., 2011). A 414 bp fragment containing the whole intergenic region *btmB-btmC*, including *btmC*_TSS_ but not its RBS, was cloned in vector pIJ10742 (Feeney et al., 2017) to create a transcriptional fusion with *gusA*. This construct was introduced in *S. scabies* WT, Δ*btmL*, and WT + L to test whether the different genetic backgrounds would affect promoter activity. As a positive control, vector pIJ10741 carrying *gusA* under the control of the constitutive promoter *ermE*^∗^p (Feeney et al., 2017) was used, while empty pIJ10742 with no promoter was employed as a negative control. *btmC*p proved to be an exceptionally strong promoter in both in solid and in liquid culture (Figure 6), and at 72 h of growth its activity was comparable to *ermE*^∗^p. This result correlates with the high levels of expression of *btmC* observed in the transcriptomic analysis. The same *gusA* reporter experiment was performed with a 545 bp region that was predicted to contain the promoter preceding *btmD*_TSS_, but no reporter activity could be detected in any of the genetic backgrounds tested (Figure 6).

Therefore, further reporter constructs were made that progressively extended the region tested until it included the whole coding sequence of *btmC* (but not *btmC*p), but these provided identical negative results (Supplementary Figures S4, S5). This result pointed to the possibility that transcription from *btmD*_TSS_ is not driven from its own promoter, but rather from *btmC*p, where some sort of secondary structure might help enhance transcription of this particular gene. To test this, we assessed the promoter activity of the whole region (*btmC*p, *btmC*, and the intergenic region before *btmD*) and compared its promoter activity to that of the original *btmC*p reporter. Two promoter versions were generated, one containing the WT sequence, and another one where several synonymous point mutations were introduced in the region of *btmD*TSS via yeast refactoring (Supplementary Figure S4) in order to disrupt possible DNA secondary structures in this region. Although these new transcriptional fusions were more active than all the other sequences lacking *btmC*p, they showed drastically reduced activity when compared to the original *btmC*p reporter, indicating that the additional DNA sequence dampened the promoter activity of *btmC*p. The mutated *btmD*TSS region did not significantly affect this phenotype (Supplementary Figures S4, S5).

### Identification of Potential Pathway Regulators Using DNA Affinity Capture Assays

Our transcriptional analysis combined with the data from *S. scabies* Δ*btmL* and WT + L show that the master regulator(s) of the *btm* cluster are not encoded by the *btm* cluster and are therefore unlikely to be pathway-specific. Identification of such regulators could aid in improving the yield of bottromycin. Given the difficulty in predicting binding sequences for transcriptional activators in streptomycetes without extensive transcriptomic and metabolic datasets (Iqbal et al., 2012; Hwang et al., 2019), the intergenic region *btmB-C* was used as a probe in a DNA-binding protein capture experiment. This region was amplified using biotinylated primers and the resulting fragment was immobilized on streptavidin coated magnetic beads. The beads were then incubated with lysates of 72 h cultures of *S. scabies* WT grown in BPM. As a negative control, the same promoter region was incubated with lysates of cultures grown in GYM medium, where the cluster is not expressed and therefore there is no bottromycin production (Supplementary Figure S6). After incubation and several washes with competitor DNA to eliminate proteins binding non-specifically to the probe, the remaining proteins were eluted, washed again and analyzed using quantitative proteomics. A total of 321 proteins were identified in the 2 sets of samples, 120 of which appeared in both conditions, whereas 177 where present exclusively in the BPM samples and only 24 were specific to GYM samples. These results were filtered to identify annotated regulatory proteins or uncharacterized proteins binding to *btmC*p specifically in bottromycin production conditions. This reduced the candidates to 21 proteins (Supplementary Table S3), of which three were annotated as regulators, while the remainder were uncharacterized proteins.

Based on the intensity of the signal obtained or the biological relevance of the hit, we selected four promising candidates for further genetic analysis to assess their involvement in bottromycin biosynthesis: MtrA (SCAB_55281), GlnK (SCAB_61751), a putative regulatory protein (SCAB_ 85931) and an uncharacterized protein that binds specifically to *btmC*p under production conditions (SCAB_51451). MtrA forms a three-component system with the histidine kinase MtrB and LpqB, a lipoprotein that is involved in signal transduction (Hoskisson and Hutchings, 2006; Nguyen et al., 2010). This system is widely conserved in Actinobacteria, where it has been shown to regulate both development and antibiotic production in *Streptomyces venezuelae* and in *Streptomyces coelicolor* (Som et al., 2017; Zhang et al., 2017). SCAB_61751 encodes a homolog of GlnK, a signal transduction protein with a central role in nitrogen metabolism (Thomas et al., 2000). SCAB_ 85931 is annotated as a putative regulatory protein and contains domains characteristic of RsbR-like anti-anti-sigma factors. Rsb proteins form part of the “stressosome,” which responds to bacterial stress and ultimately leads to the activation of the alternative sigma factor σ^B^ (Lee et al., 2004). SCAB_51451 contains no conserved domains. In order to test the role of these genes in bottromycin biosynthesis, each of them was constitutively overexpressed under the control of *ermE*^∗^p in *S. scabies* WT using the integrative plasmid pIB139-RBS, a derivative of pIB139 (Wilkinson et al., 2002). The potential involvement of the MtrAB two-component system prompted us to also express *mtrB*, which encodes the cognate histidine kinase. Despite the evidence provided by DNA binding, the bottromycin titres were comparable to the WT under the conditions tested (Supplementary Figure S7).

## Discussion

An understanding of genetic regulation is required to inform rational approaches to overproduce or engineer the biosynthesis of medicinally promising specialized metabolites. In the case of bottromycin and other RiPPs, their ribosomal origin enables the rapid generation of analogs by precursor peptide mutagenesis, which can lead to the rapid generation of libraries of bioactive analogs (Yang et al., 2018). A distinct challenge for the regulation of RiPP biosynthesis is to enable the expression of a sufficiently large amount of the precursor peptide to support RiPP production, given that this is the substrate for the pathway, while producing other pathway proteins in catalytic quantities. Numerous genetic strategies have evolved that enable this differential production of the precursor peptide. Most commonly, precursor peptide genes are encoded at the beginning of RiPP operons, where they can be followed by imperfect transcriptional terminators (sometimes called “attenuators”) that dampen transcript levels of the following genes (Severinov et al., 2007; Foulston and Bibb, 2010; O’Rourke et al., 2017). Alternatively, some RiPP gene clusters contain multiple precursor peptide genes (Li et al., 2010), while others feature a single precursor peptide containing multiple core peptides (Leikoski et al., 2013; Santos-Aberturas et al., 2019).

There is substantial interest in the RiPP antibiotic bottromycin due to its novel structure, unique molecular target and its activity toward multi-drug resistant pathogens, including methicillin-resistant *Staphylococcus aureus* (MRSA) and vancomycin-resistant enterococci (VRE) (Shimamura et al., 2009; Tacconelli and Magrini, 2017). Identification of the bottromycin biosynthetic gene cluster (Crone et al., 2012; Gomez-Escribano et al., 2012; Huo et al., 2012) showed that the precursor peptide gene, *btmD*, appeared to be encoded in the middle of a larger operon. In this study, we used 5′-tag-RNA-seq and 5′-RACE to reveal that the bottromycin gene cluster features an internal transcription start site that enhances transcription of *btmD* in relation to the remainder of the gene cluster (Figure 5B). The use of 5′-tag-RNA-seq provides confidence that this is a primary transcript and not simply the result of degradation of a longer transcript. In bottromycin production conditions, *btmD* is one of the most highly expressed genes in the genome. The presence of an internal TSS (*btmD*_TSS_) to boost precursor peptide transcript levels has not previously been reported but our data indicate that it may be a common regulatory feature in RiPP biosynthesis. Evidence for this was provided by an analysis of other RiPP gene clusters in the *S. scabies* genome, which showed that their precursor peptides also have dedicated TSSs, in some cases overlapping with the coding sequence of the preceding genes, as with *btmD.* The occurrence of intragenic TSS is not a rare phenomenon, and recent TSS mapping work in *Streptomyces clavuligerus* has revealed the presence of 155 intragenic TSSs in its genome out of a total of 2659 identified (Hwang et al., 2019) and even higher numbers have been reported for other bacteria (Wade, 2015; Brito et al., 2017).

The gene cluster encodes one putative transcriptional regulator, BtmL (Figure 1), but deletion and over-expression experiments showed that this does not function as a master regulator of biosynthesis (Figure 2) and instead functions to increase transcript levels of *btmD*. Notably, *btmD*_TSS_ is still present in *S. scabies* Δ*btmL* (Figure 5B), indicating that BtmL does not solely control this regulatory feature. Unexpectedly, a *gusA* reporter experiment did not reveal any promoter activity associated to *btmD*_TSS_, indicating that transcription from this TSS might instead be driven from *btmC*p. This could also explain why mobility shift assays with purified BtmL using this region as probe were unsuccessful (data not shown). However, a transcriptional fusion containing the entire region from *btmC*p to the region before *btmD* provided lower GusA reporter activity than only *btmC*p. This unexpected result indicates that a complex and unusual system is functioning to regulate *btmD* gene expression, and further supports the theory that *btmD*_TSS_ does not have its own dedicated promoter. These data highlight the need for further studies to elucidate the precise role of internal TSSs in RiPP biosynthesis.

Despite these null results, the importance of *btmD*_TSS_ is supported by our prior work that showed that altering the region preceding *btmD* practically abolishes bottromycin production (Eyles et al., 2018), either by deletion of *btmC* or by swapping the positions of *btmC* and *btmD* in the gene cluster. Similarly, DNA affinity capture with the *btmB-C* region did not pull down BtmL (Supplementary Table S3). Therefore, the mechanism by which BtmL modulates *btmD* transcription remains cryptic. One possible hypothesis would be the interaction with a *cis*-regulatory region in the 5′ untranslated region (UTR) of *btmD* mRNA. Transcripts with UTRs bigger than 100–150 bp are usually considered “extended-leadered mRNAs” and are likely candidates to contain such a regulatory feature (Brito et al., 2017; Hwang et al., 2019). The *btmD* 5’UTR (347 bp) is within this category, but searches in this sequence against the Rfam database for any known RNA structures did not yield a clear result.

We sought to use our knowledge of pathway expression to increase bottromycin titre, firstly by over-expressing *btmL* either alone or along with *btmA*, which encodes a MFS transporter. While this increased *btmD* transcription (Figure 2B), it did not lead to higher bottromycin yields, indicating that *btmD* levels are not the rate limiting factor for pathway expression. Similarly, in *S. scabies*, export by BtmA is not a bottleneck in this pathway. We had previously shown that the bottromycin pathway produces a significant amount of side-products in both *S. scabies* and a heterologous expression system (Crone et al., 2016; Eyles et al., 2018). Pathway refactoring in this heterologous system showed that constitutive high expression of the *btm* cluster leads to high yields of shunt metabolites and low titres of mature products, while controlled expression using a riboswitch increases overall production with higher ratios of mature bottromycins versus shunt metabolites. This is consistent with a study that refactored the bottromycin gene cluster from *Streptomyces* sp. BC16019 (Horbal et al., 2018), which also concluded that high levels of transcription did not fully correlate with increases in production. Promoter choice is a key factor for optimizing production of RiPPs, and can also depend on the timing of gene expression, such as in the heterologous production of telomestatin in *Streptomyces avermitilis* (Amagai et al., 2017).

There is surprisingly little known about the regulation of specialized metabolites that do not contain master regulators in their gene clusters. A rare example is erythromycin biosynthesis in *Saccharopolyspora erythraea*, which is regulated by BldD, a key regulator of actinomycete development, as well as two other regulatory genes which are not located within its biosynthetic cluster (Chng et al., 2008; Kirm et al., 2013; Wu et al., 2014). We sought to identify the master regulators of bottromycin biosynthesis using a DNA affinity capture experiment with the intergenic region between *btmB* and *btmC*, which contains the major promoters that control the pathway. This provided a series of promising candidate proteins, including the well-characterized regulatory proteins MtrA and GlnK. However, over-expression of these genes also did not lead to increased bottromycin production in *S. scabies*. This negative result does not disprove the involvement of these regulators, in particular in the case of MtrA and GlnK. As a member of classical two-component system the activity of the response regulator MtrA is dependent on its phosphorylation state (Kenney, 2002; Desai and Kenney, 2017). Overexpression of the gene, if it is not appropriately phosphorylated, might not be enough to reveal a phenotype. GlnK is a PII family signal transducer protein that modulates the activity of multiple biosynthetic and regulatory proteins in nitrogen metabolism (Arcondéguy et al., 2001; Gerhardt et al., 2015; Shimizu, 2016). It would make sense that nitrogen levels would exert an effect on bottromycin biosynthesis, due to the peptidic nature of bottromycin and in fact a paralog of this protein has been shown to affect secondary metabolite production in *S. coelicolor* (Waldvogel et al., 2011). These proteins also rely on post-translational modifications to properly function, which adds a layer of complication in their regulatory functions that may dampen the effect of their overexpression. Further studies will be necessary to fully characterize the role of these regulators, as well as the uncharacterized proteins identified in this work, on bottromycin production.

In summary, we have revealed that the regulation of bottromycin biosynthesis is surprisingly complex and features interplay between global regulatory proteins and a cluster-situated modulator, BtmL. Precursor peptide transcription is enhanced in relation to surrounding genes by BtmL and a cryptic internal transcriptional start site. This single-gene internal transcript makes the precursor peptide gene one of the most highly transcribed genes in the *S. scabies* genome. A wider assessment of the *S. scabies* genome shows that this strategy could represent a widespread regulatory mechanism for the expression of RiPP precursor peptide genes.

## Materials and Methods

### Chemicals and Molecular Biology Reagents

Unless otherwise specified, antibiotics, media components and chemical reagents used in this work were purchased from Sigma-Aldrich (United Kingdom) with the exception of soy flour, which was purchased from Holland & Barret (United Kingdom). Enzymes and molecular biology kits were purchased from New England Biolabs and Promega Healthcare, respectively.

### Bacterial Strains and Culture Conditions

The following strains were used in this work: *S. scabies* DSM 41658, both the wild-type (WT) and the mutant strain Δ*btmL* (ΔL), whose construction was described previously (Crone et al., 2012). Additionally, *Escherichia coli* K-12 strain DH5α (Invitrogen) was used for plasmid propagation and DNA manipulation, and the methylation deficient strain *E. coli* ET12567 containing pUZ8002 (Paget et al., 1999) was used for intergeneric conjugal transfer of genetic material to *S. scabies*, which was performed following standard procedures (Kieser et al., 2000). *E. coli* culture media used in this work are described in Sambrook et al. (1989). Several *Streptomyces* culture media were used: mannitol soya flour medium (SFM) (Kieser et al., 2000) was used for *Streptomyces* propagation and conjugations, and instant potato mash agar [2% Smash (Premier Foods), 2% agar] was used to grow *S. scabies* for spore harvesting. GYM medium (0.4% glucose, 0.4% yeast extract, and 1.0% malt extract, in Milli-Q water) and bottromycin production medium (BPM: 1% glucose, 1.5% soluble starch, 0.5% yeast extract, 1.0% soy flour, 0.5% NaCl, and 0.3% CaCO_3_, in Milli-Q water) were used for bottromycin production. Production experiments were performed as follows: 30 μL of concentrated spores were used to inoculate 10 mL of GYM medium in 50 mL flasks and were incubated for 48 h at 30°C and 250 rpm. 250 μL of seed culture were used to inoculate 10 mL BPM in 50 mL Falcon tubes covered with foam bungs instead of caps. Alternatively, 1 mL of seed culture was used to inoculate 50 mL of BPM in 250 mL flasks containing a spring. When necessary, cultures were supplemented with appropriate concentrations of CoCl_2_ (25 μg/mL unless otherwise stated). Triplicate production cultures were incubated for 5–6 days at 28°C and 230 rpm, at which point samples were collected and processed immediately, or frozen at −20°C until further processing and analysis. When antibiotic selection was necessary, culture media were supplemented with the appropriate antibiotics at the following final concentrations: kanamycin at 50 μg/mL, apramycin at 50 μg/mL, hygromycin at 50 μg/mL, chloramphenicol at 25 μg/mL, and nalidixic acid at 25 μg/mL.

### Gene Expression Plasmids Construction

All the primers used for gene amplification and generation of the following constructs are listed in Supplementary Table S1. The gene expression vectors used in this work were pIB139-RBS-btmD (Crone et al., 2016) and pIJ10257 (Hong et al., 2005), which are both integrative plasmids containing the constitutive *ermE*^∗^ promoter. pIB139-RBS-btmD is a derivative of pIB139 (Wilkinson et al., 2002) that carries the *btmD* gene preceded by a ribosome binding site (RBS) and an *Nde*I restriction site installed to facilitate cloning and improve gene expression (Crone et al., 2016). This plasmid was linearized using restriction enzymes *Nde*I and *Eco*RI, releasing *btmD* and allowing the introduction of the gene of interest, either via ligation of an *Eco*RI/*Nde*I treated PCR fragment or by Gibson assembly, following published protocols (Crone et al., 2012). The ligation method was used to introduce *btmL* and generate pIB139-RBS*-btmL*, which was used to complement the mutant Δ*btmL* and to overexpress this gene in the WT strain (WT + L). The Gibson assembly method was used to introduce *mtrA*, *mtrB*, *glnB*, SCAB_85931, and SCAB_51451, into pIB139-RBS for gene overexpression the WT strain. An empty version of pIB139-RBS was generated to use as a control in production experiments, via Gibson assembly, using the *Nde*I/*Eco*RI linearized vector and primers pIB-RBS_fw and pIB-RBS_rv, carrying the RBS sequence. pIJ10257 was used for the expression of *btmA*. This gene was amplified using primers btmA-start and btmA-end, digested with *Nde*I and *Hin*dIII and ligated into *Nde*I/*Hin*dIII digested pIJ10257. Correct construction of all expression plasmids was confirmed using colony PCR and sequencing, after which they were transferred to *E. coli* ET12567/pUZ8002 cells for conjugation into *S. scabies* (WT or Δ*btmL* as appropriate). pIB139 integrates in the ϕC31 phage integration site, and pIJ10257 integrates in the ϕBT1 site, which allowed for the simultaneous overexpression of two genes when necessary. Correct integration of the plasmids was verified by colony PCR using ermEp_chk1 in combination with the reverse amplification primer for each of the genes. As a control for the production experiments, a strain carrying the corresponding empty vector was generated in each case.

### LC-MS Analysis of Bottromycin Production

Bottromycin production culture samples (1 mL) were extracted with an equal volume of methanol, mixed with shaking for a minimum of 10 min. The mixtures were then centrifuged for 4 min at 13,000 rpm to pellet cellular material and other particulate contaminants. 2 μL of the resulting supernatants were injected onto a Phenomenex Kinetex 2.6 μm XB-C18 column (50 mm × 2.1 mm, 100 Å) attached to a Shimadzu Nexera X2 UHPLC and eluted with a linear gradient of 5 to 95% acetonitrile (ACN) in water + 0.1% formic acid (FA) over 6 min, with a flow-rate of 0.6 mL/min. MS data were obtained in positive mode using a Shimadzu IT-TOF mass spectrometer coupled to the UHPLC and analyzed using LabSolutions software (Shimadzu). Bottromycin production was plotted in peak area units. To normalize production values across samples, culture growth was quantified by measuring DNA concentration with an adaptation of the Burton diphenylamine colorimetric assay (Zhao et al., 2013).

### Isolation of Total RNA

Two milliliter samples of *S. scabies* liquid cultures were harvested after 72 h incubation in conditions as described above. Samples were washed with an equal volume of RNAlater (Thermo Fisher Scientific) and stored at −80°C until further processing. RNA was then extracted following previous protocols (Crone et al., 2016), resuspending the mycelium in 1 mL of RLT buffer from the RNeasy Kit (Qiagen) and homogenizing the sample in lysing matrix B tubes (MP Biomedicals) using a FastPrep instrument and a program of 3 × 30 s pulses at 6 m/s with 1 min cooling intervals on ice. The lysates were then centrifuged at 13,000 rpm and 700 μL of supernatant from each sample were then transferred to spin tubes from the RNeasy Kit to undergo purification following manufacturer instructions. Chromosomal DNA contamination was eliminated with on-column DNase I treatment (Qiagen) and a further cleanup step using TURBO DNA-free Kit (Ambion, Invitrogen). RNA concentration in the samples was quantified measuring *A*_260_ using a Nanodrop. In the case of the samples for 5′tag-RNAseq, further quantity and integrity measurements were performed with RNA ScreenTape (Agilent).

### RT and qRT-PCR Analyses

Both RT-PCR and qRT-PCR analyses were carried out using 250 ng of total RNA as template in a two-step protocol. The first step consisted of cDNA synthesis with the QuantiTect Reverse Transcription Kit (Qiagen), as detailed in the manufacturer’s instructions. For RT-PCR analyses the cDNA was then used as a template for PCR reactions using Taq polymerase and specific primers for the regions to test (Supplementary Table S1). The amplification conditions were as follows: initial denaturation at 95°C for 3 min followed by 33 cycles of 95°C for 30 s, 58–62°C for 30 s and 72°C for 40–60 s, with a final extension step at 72°C for 5 min. The resulting RT-PCR products were separated in 2% agarose gels and stained with ethidium bromide for visualization. For qRT-PCR analyses the aforementioned cDNA was used as template in quantitative reactions with the SensiFAST SYBR No-ROX Kit (Bioline) following the manufacturer’s instructions. The reactions were run in a Bio-Rad CFX96 thermocycler and the amplification protocol was a 2-step cycling PCR program: 1 cycle at 95°C for 2 min followed by 40 cycles of 5 s at 95°C and 30 s at 60°C. An additional melting curve step was used at the end of the reaction to assess the specificity of the amplified products. The qRT-PCR results were analyzed with CFX Manager software (Bio-Rad). In both cases, negative control samples of the cDNA synthesis step with no retrotranscriptase were included, in order to control for the presence of contaminating chromosomal DNA in the RNA samples. Primers used in these analyses (Supplementary Table S1) were designed with a preference for 17–23 mers and *T*_m_ ∼ 65°C with the help of Vector NTI Advance 11.5 (Invitrogen) and Primer3 software (Untergasser et al., 2012) and validated used the online tool NetPrimer (Premier Biosoft). In the specific case of qRT-PCR primers, these were designed to amplify fragments of ∼100 bp and their efficiency was tested using serial dilutions of chromosomic DNA as template. For both RT-PCR and qRT-PCR, primers for *hrdB*, encoding a housekeeping sigma factor, were used as an internal control to assess the quality of RNA and in the latter case to normalize gene expression levels.

### 5′tag-RNA-Seq (tagRNA-Seq)

#### Library Construction and Sequencing

Total RNA samples of *S. scabies* WT and Δ*btmL* were extracted as previously described and, following quality control assays to ensure their integrity, they were submitted to Vertis Biotechnologie AG (Germany) for the construction and sequencing of tagRNA-seq libraries in a protocol adapted from the technique described in Innocenti et al. (2015). Prior to library construction, rRNA was depleted in the samples using the Ribo-Zero rRNA Removal Kit for bacteria (Epicenter). The remaining material was labeled sequentially as follows: sequence tag CTGAAGCT was ligated to transcripts presenting 5′-monophosphate groups (processed transcripts). The samples were then treated with RNA 5′ polyphosphatase (5′PP; Epicenter) to convert the 5′-triphosphate groups of primary transcripts into 5′-monophosphate ends amenable for ligation with the alternative tag sequence TAATGCGC. Once labeled, the samples were used as template for first-strand cDNA synthesis using random hexameric primers. After fragmentation and RNA clean up, Illumina TruSeq sequencing adapters were ligated in a strand specific manner to the 5′ and 3′ ends of the cDNA fragments. The cDNA was then amplified with a proof-reading enzyme to enrich the samples. At this point, it is possible to specifically PCR amplify the 5′-ends which carried the two tag sequences, but that would mean losing the information relating to the rest of the transcriptome. Therefore, a full transcriptome enrichment (for fragments that had Illumina adapters on both ends) was carried out in order to preserve the full coverage of the transcriptome in our libraries. The resulting material was purified with the Agencourt AMPure XP Kit (Beckman Coulter Genomics) and analyzed by capillary electrophoresis. The cDNA preparations were pooled in equimolar amounts and size selected (240–450 bp range) and the pooled libraries were sequenced on an Illumina NextSeq 500 system using 75 bp read lengths. RNA-seq data has been deposited in the ArrayExpress database (accession number E-MTAB-8236).

#### Data Analysis

Sequencing reads were first sorted according to their tags in order to generate independent fastq files for each of the datasets (see Supplementary Table S2 for a summary of the sequencing results). Once sorted, the reads were trimmed to remove the tag sequences and aligned to *S. scabie*s 87-22 genome (RefSeq NC_013929.1) using Bowtie2 (Langmead and Salzberg, 2012), which yielded SAM alignment files for each of the fastq files. Downstream processing of the SAM alignment files was performed using a series of Perl scripts supported by the Bioperl (Stajich et al., 2002) toolkit. Using the SAM alignments as input, the number of reads mapping to each nucleotide position of the *S. scabies* genome was calculated and saved in a coverage file. The coverage information was then integrated with a feature table including the coordinates of all of the gene coding sequences in the genome, in order to calculate the number of reads per gene, or gene counts. These gene counts were used to make an estimation of gene expression levels by calculating RPKM and TPM values for each gene (Wagner et al., 2012) (Supplementary Datasheet S1). For mapping and visualization of the transcription start sites (TSSs) in the bottromycin cluster, the SAM files containing the sequences labeled with 5′ tags were transformed into wig files suitable for visualization in the Integrated Genome Browser (IGB) software (Freese et al., 2016) plotted against the *S. scabies* genome. These files were normalized to eliminate biases due to difference in sequencing depth using the normalizeQuantiles function in the limma package (Ritchie et al., 2015) of R (R Core Team, 2014). Given the high amount of processed transcripts present in the *btmC-D* region of the bottromycin cluster, a “wig minus wig” file was generated in which processed transcript reads were subtracted from the primary transcript reads. The regions mapped in this filtered file were considered to be true TSSs.

### 5′-RACE Experiments

The TSSs identified in the 5′tag-RNA-seq experiment were validated using a 5′-RACE system for rapid amplification of cDNA ends (Invitrogen), using the manufacturer’s instructions (version 2.0). 1 μg of total RNA from *S. scabies* WT and Δ*btmL* harvested after 72 h of growth at 28°C and 230 rpm was used to carry out cDNA synthesis with specific primers for each of the promoters tested (Supplementary Table S1). The cDNA was purified and treated with terminal deoxynucleotidyl transferase (TdT) to add poly(dC) tails to its 3′ends. After an initial PCR amplification of the tailed fragments with the 5′-RACE abridged anchor primer and subsequent amplifications with the universal amplification primer (both provided by the kit) and specific nested primers (Supplementary Table S1), defined amplification products were observed. These products were gel purified and submitted for Sanger sequencing to confirm the position of the TSSs.

### *gusA* Transcriptional Fusions Construction and β-Glucuronidase Reporter Activity Assays

Putative promoter regions of *btmC* (*btmC*p, 414 bp) and *btmD* (*btmD*p, 545 bp) and extended promoter regions spanning different lengths of *btmC* were amplified from *S. scabies* genomic DNA or from a refactored bottromycin cluster containing 33 synonymous point mutations in the *btmD*_TTS_ region (Supplementary Figure S4) with primers containing *Nde*I and *Xho*I restriction sites (Supplementary Table S1). These were verified by sequencing, and then ligated or assembled into *Nde*I/*Xho*I digested pIJ10742, which contains a promoterless copy of the reporter gene *gusA* (Feeney et al., 2017). These plasmids were introduced via intergeneric conjugation into *S. scabies* (WT, Δ*btmL*, and WT + L) using *E. coli* ET12567/pUZ8002, where they integrated in the ϕBT1 phage integration site. Hygromycin resistant exconjugants were analyzed by colony PCR with primers pGUS_chk_fw and pGUS_chk_fw. β-glucuronidase assays in solid and liquid medium were carried out as described previously (Sherwood and Bibb, 2013). In the case of the liquid assays, reporter activity was represented as Miller units/mg protein. In both cases, pIJ10742 carrying no promoter was used as a negative control, and pIJ10741, carrying *gusA* under the control of *ermE*^∗^p (Feeney et al., 2017), was included as a positive control of promoter activity.

### DNA Affinity Protein Capture Assay

#### Sample Preparation

The intergenic region *btmB-btmC* (414 bp) was PCR amplified using primers probe_BC_fw2_b and probe_BC_rv2 to generate a 5′ biotinylated probe. 40 μg of this probe were inmobilised onto 10 mg of streptavidin magnetic beads (Dynabeads^®^ MyOne^TM^ Streptavidin T1, Invitrogen, United Kingdom), following the manufacturer’s instructions. Protein extracts from *S. scabies* wild type were obtained from 500 mL cultures in either BPM or GYM incubated for 72 h at 28°C and 250 rpm. Cell pellets were harvested by centrifugation for 15 min at 7,000 rpm, resuspended in binding buffer (20 mM TrisHCl pH7.5, 1 mM EDTA, 100 mM NaCl, 10% glycerol, and 1 mM DTT) supplemented with protease inhibitors (cOmplete^TM^ Protease Inhibitor Cocktail, Roche, United Kingdom) and lysed by sonication with a Vibra-cell sonicator (Sonics & Materials Inc., United States) using 150 × 2 s pulses at 40% amplitude alternated by 5 s rest on ice. Binding assays were performed using a modification of the protocol reported by Bekiesch et al. (2016) using 60 mg of total protein in a 10 mL final volume of the aforementioned binding buffer supplemented with 0.1 mg/mL of salmon sperm DNA (Invitrogen, Germany). Proteins were eluted twice in 250 μL binding buffer containing 2 M NaCl and the binding assay was repeated twice for each sample, generating 1 mL of final eluate per sample. These eluates were then acidified to pH3 with trifluoroacetic acid (TFA) and applied to an C4 SPE column (OMIX C4, Agilent). The samples were washed twice with 0.1% TFA in water and three times with 0.1% FA before eluting in three fractions (200 μL 30% ACN and 0.1% FA, 200 μL 30% ACN and 200 μL 70% ACN) that were pooled and kept frozen on dry ice until analysis.

#### Sample Analysis

After purification and clean up, duplicate samples for each condition were analyzed by LC-MS/MS on an Orbitrap-Fusion^TM^ mass spectrometer (Thermo Fisher, United Kingdom) equipped with an UltiMate^TM^ 3000 RSLCnano System using an Acclaim PepMap C18 column (2 μm, 75 μm × 500 mm, Thermo). Samples were loaded and trapped using a pre-column which was then switched in-line to the analytical column for separation. Peptides were eluted with a gradient of 5–40% ACN in water/0.1% FA at a rate of 0.5% min^–1^. The column was connected to a 10 μm SilicaTip^TM^ nanospray emitter (New Objective, United States) for infusion into the mass spectrometer. Data dependent analysis was performed using a CID/HCD fragmentation method with the following parameters: positive ion mode, orbitrap MS resolution = 60 k, mass range (quadrupole) = 300–1500 *m/z*, AGC target 2e^5^, MS2 in ion trap, threshold 1e^4^, isolation window 1.6 Da (quadrupole), charge states 2–6, MS2 top20, AGC target 1.5e^4^, max inject time 200 ms, dynamic exclusion 1 count, 60 s exclusion, exclusion mass window ±7 ppm. MS scans were saved in profile mode while MS2 scans were saved in centroid mode. Raw files were processed with MaxQuant (version 1.5.3.30) (Tyanova et al., 2016). The peak lists were searched against a *S. scabies* protein database downloaded from Uniprot.org (25.05.2016) with 16846 entries together with the MaxQuant contaminants database (249 entries) using an in-house Mascot Server (2.4.1, Matrix Science, United Kingdom) with trypsin with 2 missed cleavages, carbamidomethylation (C) as fixed and oxidation (M), acetylation (protein N-terminus), and deamidation (N,Q) as variable modifications. Mass tolerances were 6 ppm for precursor ions and 0.6 Da for fragment ions. Mascot search results were imported into the Scaffold software (Proteome Software Inc., United States) to probabilistically validate protein identifications derived from the MS/MS sequencing results using the X!Tandem (Craig and Beavis, 2003) and ProteinProphet algorithms (Nesvizhskii et al., 2003). Validation parameters were set to 95% protein probability and 95% peptide probability.

## Data Availability Statement

The datasets generated for this study can be found in the ArrayExpress Archive of Functional Genomics Data (accession number E-MTAB-8236).

## Author Contributions

NV performed genetic manipulation experiments, LC-MS analyses, RNA extraction, and gene expression analyses. NV, EC-T, and TE performed promoter activity assays. GC and NV performed RNA-seq data processing and analysis. AT devised and supervised this work. NV and AT wrote the manuscript.

## Conflict of Interest

The authors declare that the research was conducted in the absence of any commercial or financial relationships that could be construed as a potential conflict of interest.
