# Supplementary material for: Regulation of Bottromycin Biosynthesis Involves an Internal Transcriptional Start Site and a Cluster-Situated Modulator
Source: Front Microbiol. 2020 Mar 26;11:495. doi: 10.3389/fmicb.2020.00495 (PMC7113386; doi:10.3389/fmicb.2020.00495)
Supplement: Supplementary file 2 [file Data_Sheet_2.pdf]

# *Supplementary Material*

1 **Supplementary table S1.** Primers used in this work.

2 **Supplementary Figure S1.** Temporal progression of the expression of the *btm* cluster in bottromycin production medium (BPM).

3 **Supplementary Table S2.** Summary of RNA-seq output.

4 **Supplementary Figure S2.** Transcription start site mapping of putative RiPP precursor peptides in *S. scabies* using 5'-tag-RNA-seq.

5 **Supplementary Figure S3.** Transcription start site mapping of putative RiPP precursor peptides in *S. scabies* using 5'-tag-RNA-seq (continuation).

6 **Supplementary Figure S4.** Promoter activity of *btmD*p variants using β-glucuronidase (GUS) reporter assays in solid medium.

7 **Supplementary Figure S5.** Quantitative analysis of promoter activity of *btmD*p variants using β-glucuronidase (GUS) reporter assays in liquid medium.

8 **Supplementary Figure S6.** RT-PCR analysis of the cultures used for the DNA affinitiy capture assay.

9 **Supplementary Table S3.** Filtered list of proteins captured in the DNA affinity pull-down experiment that bind to *btmC*p in bottromycin production conditions.

10 **Supplementary Figure S7.** Effect of potential transcriptional activators of the bottromycin pathway on antibiotic production.

**Supplementary Table S1.** Primers used in this work. Restriction sites used for cloning or assembly are underlined and in bold type. The RBS sequence introduced during the construction of pIB-RBS appears in bold and grey background.

| Primer name | Sequence (5'-- 3') | Use |
|---|---|---|
| btmA-start | CCCGACCAGAATGCC**CATATG**CTGC | Amplification and cloning of *btmA* in pIJ10257 |
| btmA-end | TGCGG**AAGCTTA**CGCGGACAGTCTG | |
| pIB-RBS fw | TAGGATCCACATAATAA**AGGAGG**ACCAC**CATATG**TTGGGGATCCTCTAGAGGATCCGCGGCCGCGCGCGATATC**GAATTC**GTAATCATGTCATAGCTGTTTCCTG | Generation of vector pIB-RBS (empty pIB139 derived vector carrying a canonical RBS, highlighted in the sequence) |
| pIB-RBS rv | CAGGAAACAGCTATGACATGATTAC**GAATTC**GATATCGCGCGCGGCCGCGGATCCTCTAGAGGATCCCCAA**CATATG**GTGGT**CCTCCT**TTATTATGTGGATCCTA | |
| mtrA-start | TAGGATCCACATAATAAAGGAGGACCAC**CATATG**GCATTCATGAAGGGAC | Amplification and assembly of *mtrA*, *mtrB*, *glnB*, *SCAB_85931* and *SCAB51451* in pIB-RBS |
| mtrA-end | ACAGGAAACAGCTATGACCATGATTAC**GAATTC**ACCGGACATCTCAGCTG | |
| mtrB-start | TAGGATCCACATAATAAAGGAGGACCAC**CATATG**TCCGGTGACAGTGCC | |
| mtrB-end | ACAGGAAACAGCTATGACCATGATTAC**GAATTC**CGCACGGTCAGCGCCC | |
| glnB-start | TAGGATCCACATAATAAAGGAGGACCAC**CATATG**AGACTCATCACCGCG | |
| glnB-end | ACAGGAAACAGCTATGACCATGATTAC**GAATTC**CGTGCGCTCAGAGCGC | |
| SCB85931-start | TAGGATCCACATAATAAAGGAGGACCACCATGTGCCGGAACAGGACACG | |
| SCB85931-end | ACAGGAAACAGCTATGACCATGATTAC**GAATTC**GCCGCTCACCTGTGCC | |
| SCB51451-start | TAGGATCCACATAATAAAGGAGGACCAC**CATATG**GCCGCAAGGCCGCTC | |
| SCB51451-end | ACAGGAAACAGCTATGACCATGATTAC**GAATTC**CCAGTACCCCGTCCGCG | |
| ermEp_chk1 | CGATCTTGACGGCTGGCGAG | PCR verification of exconjugants |
| qhrdBs fw | CGTGGAGAACTTGTAGCCCTTGG | qRT-PCR analysis of the *btm* cluster expression |
| qhrdBs rv | CATGCTCTTCCTGGACCTGATCC | |
| qRT-A fw | GATGTCCGGTGCGAGGGCT | |
| qRT-A rv | CCGTCGTCTGGTTCGTCGTCC | |
| qRT-B fw | CGATCAGCCGCAACCCG | |
| qRT-B rv | ACCCGCTCCCTGACCCTCTT | |
| qRT-C fw | CGATGAGAAGTGGGCGGAGG | |
| qRT-C rv | CCGGCGGCACGAGAAGAT | |
| qRT-D fw | CGGACTTCCTCAACGACGACC | |
| qRT-D rv | GGCTTCACCGTCCCAGGC | |
| qRT-E fw | ACCCGTCTCTGCTTCGCCC | |
| qRT-E rv | GGTCCTCCTCACTTCTCCCGT | |
| qRT-L fw | CGCAGAGGCCAAGGTCAACG | |
| qRT-L rv | GTCGCGCCGCAGCAGCT | |

| | | |
|---|---|---|
| RT-AB fw | TGTTGCCCAGCAGGGACAGG | RT-PCR analysis of *btm* cluster intergenic regions |
| RT-AB rv | GCCACATCTCCCGCTTCGC | |
| RT-CD fw | TCGTCCCCCCGTACCTCTCA | |
| RT-CD rv | GCTCAGCTCCGCGTTGTTGG | |
| RT-DE fw | GACCCCAACAACGCGGAGC | |
| RT-DE rv | GAGGGATACGAACGGTGGACGAC | |
| RT-KL fw | CGCTCACGCCCACGCATT | |
| RT-KL rv | CAGATCGCGGCAGGACGG | |
| RT_hrdB_fw | GGACCTTGCCGATCTGCTTGA | |
| RT_hrdB_rv | GGGGAAAGGCTGAGGGGCA | |
| btmDp_GSP1 | GCTTCCGACAGGGCTTTC | 5' RACE verification of transcription start sites for *btmB*, *btmC* and *btmD* |
| btmDp_GSP2 | GTGCCACGGCCTCGGTCGT | |
| btmDp_nested | CCT CGG TCG TGG CGG TGC | |
| btmDp_nested2 | CGAGGGATACGAACGGTGGACG | |
| btmCp_GSP1 | GGCTGAACATCGAGGTGAA | |
| btmCp_GSP2 | GGGGGCGGACTCCAGCAA | |
| btmCp_nested | CGGTGTCCTCCACATGACGGT | |
| btmBp_GSP1 | CGGAGAGGTACATGCTGAG | |
| btmBp_GSP2 | CCACGAGGACGGGCCGGT | |
| btmBp_nested | CAGTCGGGTTCGGTGACGGA | |
| btmBp_nested2 | CGGGATGTGCGTCGAGAAATG | |
| probe_btmDp_fw | **CATATG**ACTTCACCGACGACCCCG | Construction of transcriptional fusions for β-glucuronidase (GUS) reporter activity assays |
| probe_btmDp_rv | **CTCGAG**TCTGTCTTCCGGGTCGC | |
| probe_btmDp_fw-1 | **CATATG**AACATCCGGTTCCTGCTCG | |
| probe_btmDp_rv-2 | **CTCGAG**AGCGGTGAGCGTGAGAGG | |
| probe_btmDp_rv-1 | **CTCGAG**CTCTTGCCTCAGAGGGACG | |
| probe_BC_fw2 | **CATATG**GAAATCTTCACGCGACCTC | |
| probe_BC_rv2 | **CTCGAG**CCGATTGCGGCAACT | |
| probe_BC_fw2_tail | TCCCCGGGAATTCTCTAGAGGATC**CATATG**GGAAAT CTTCACGCGACCTC | |
| probe_btmDp_fullC5'_fw | TCCCCGGGAATTCTCTAGAGGATC**CATATG**GGCTCC GGGGGTTGATTT | |
| probe_btmDp_fullC5'_rv | CGGTGAGGATGTTCCAGTCGG | |
| probe_btmDp_rv_tail | CATGTCCGTACCTCCGTTGCTCGA**CTCGAG**GTCTGT CTTCCGGGTCGC | |
| pGUS_chk_fw | GCTTGCTCAATCAATCACCG | PCR verification of GUS constructs |
| pGUS_chk_rv | ACCGCTGGTCGATACCGC | |
| pGUS_chknew_fw | GATCCCCGGGAATTCTCTAGAGG | |
| probe_BC_fw2_b | **Biotin-**GGAAATCTTCACGCGACCTC | Generation of biotinylated probe for capture assay |

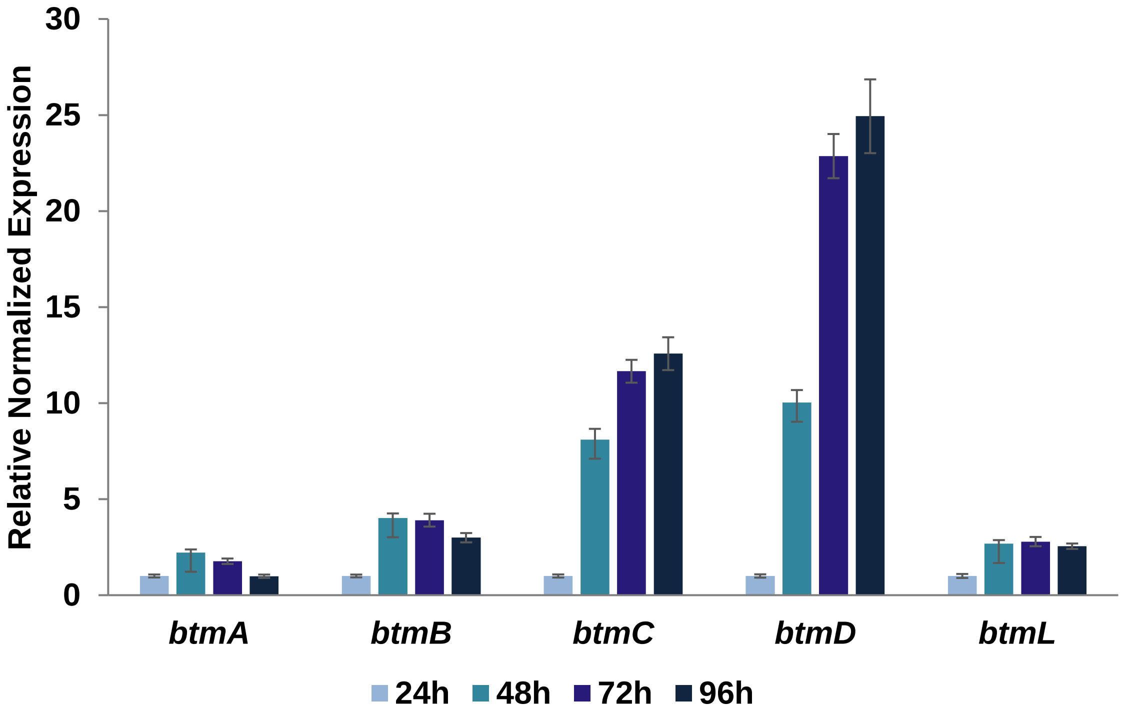


**Supplementary Figure S1.** Temporal progression of the expression of the *btm* cluster in bottromycin production medium (BPM). qRT-PCR analysis of the transcription of representative *btm* genes in *S. scabies* WT after 24, 48, 72 and 96 h growth in BPM. Expression values are relative to the expression of the target gene at 24 h post inoculation, which was set at 1. Transcription of *hrdB* was used to normalize the expression levels across samples. Error bars represent standard error of the mean from triplicate experiments.

**Supplementary Table S2.** Summary of RNA-seq output. Total number and percentage of RNA-seq reads carrying either no tag or one of the two specific tags used to determine transcription start sites (TSS) and processing sites (PS) in the transcriptomes of *S. scabies* WT and the mutant *ΔbtmL.*

| | Strain | | | |
|---|---|---|---|---|
| | WT | | *ΔbtmL* | |
| | Number of reads | % of total | Number of reads | % of total |
| **No tags found (internal transcripts)** | 26,657,607 | 82.0 | 24,271,685 | 84.9 |
| **CTGAAGCT tag (PS transcripts)** | 2,071,620 | 6.4 | 1,151,077 | 4.0 |
| **TAATGCGC tag (TSS transcripts)** | 3,767,782 | 11.6 | 3,161,326 | 11.1 |
| **Total** | 32,497,009 | 100 | 28,584,088 | 100 |

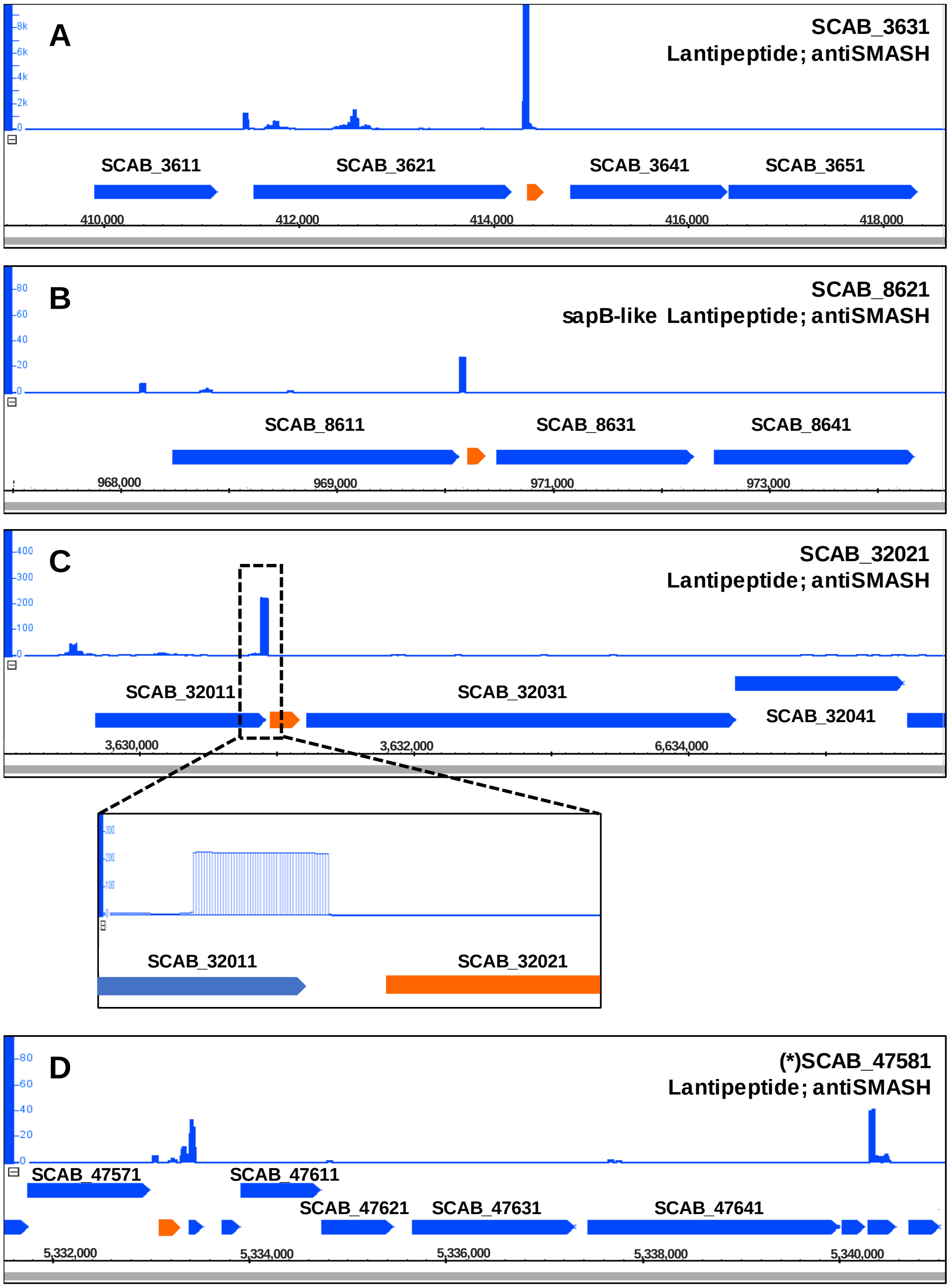


**Supplementary Figure S2.** Transcription start site mapping of putative RiPP precursor peptides in *S. scabies* using 5'-tag-RNA-seq. Each of the plots (generated in IGB) represents a putative cluster predicted by antiSMASH and is labelled with the locus tag of the potential precursor peptide gene (also highlighted in orange in the map), the predicted RiPP product and the tool used to identify it. The detail in panel C shows the precise mapping of SCAB_32021 TSS within the coding sequence of the previous gene. (*) SCAB_47581 is no longer annotated in the latest version of *S. scabies* genome.

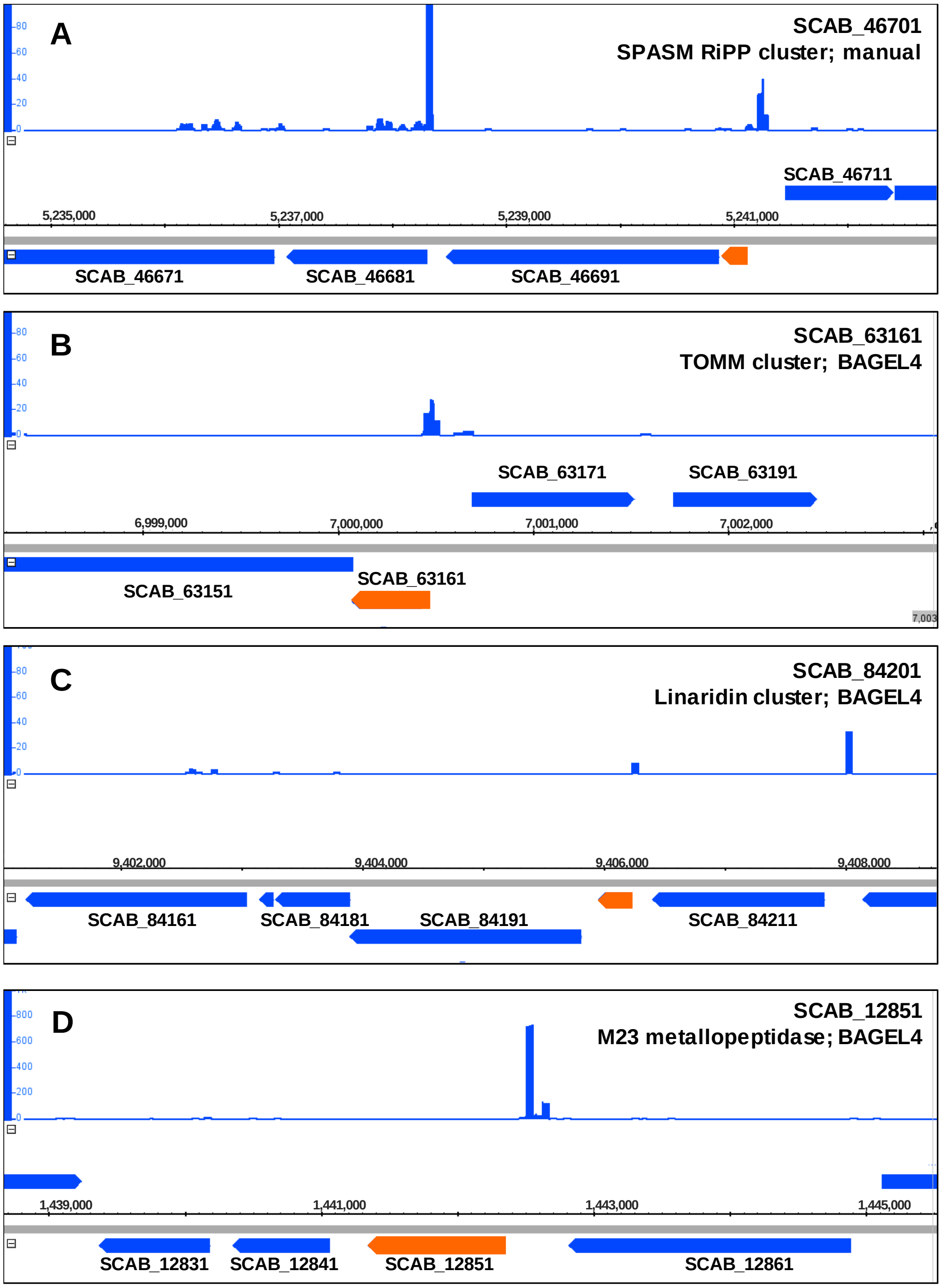


**Supplementary Figure S3.** Transcription start site mapping of putative RiPP precursor peptides in *S. scabies* using 5’-tag-RNA-seq (continuation). Plots A, B and C represent putative RiPP clusters predicted either manually or using BAGEL4 and are labelled with the locus tag of the potential precursor peptide gene (also highlighted in orange in the map), the predicted RiPP product and the prediction tool used to identify it. Panel D depicts the TSS for a M23 family metallopeptidase gene predicted by BAGEL4, (highlighted in orange) rather than a RiPP precursor peptide.

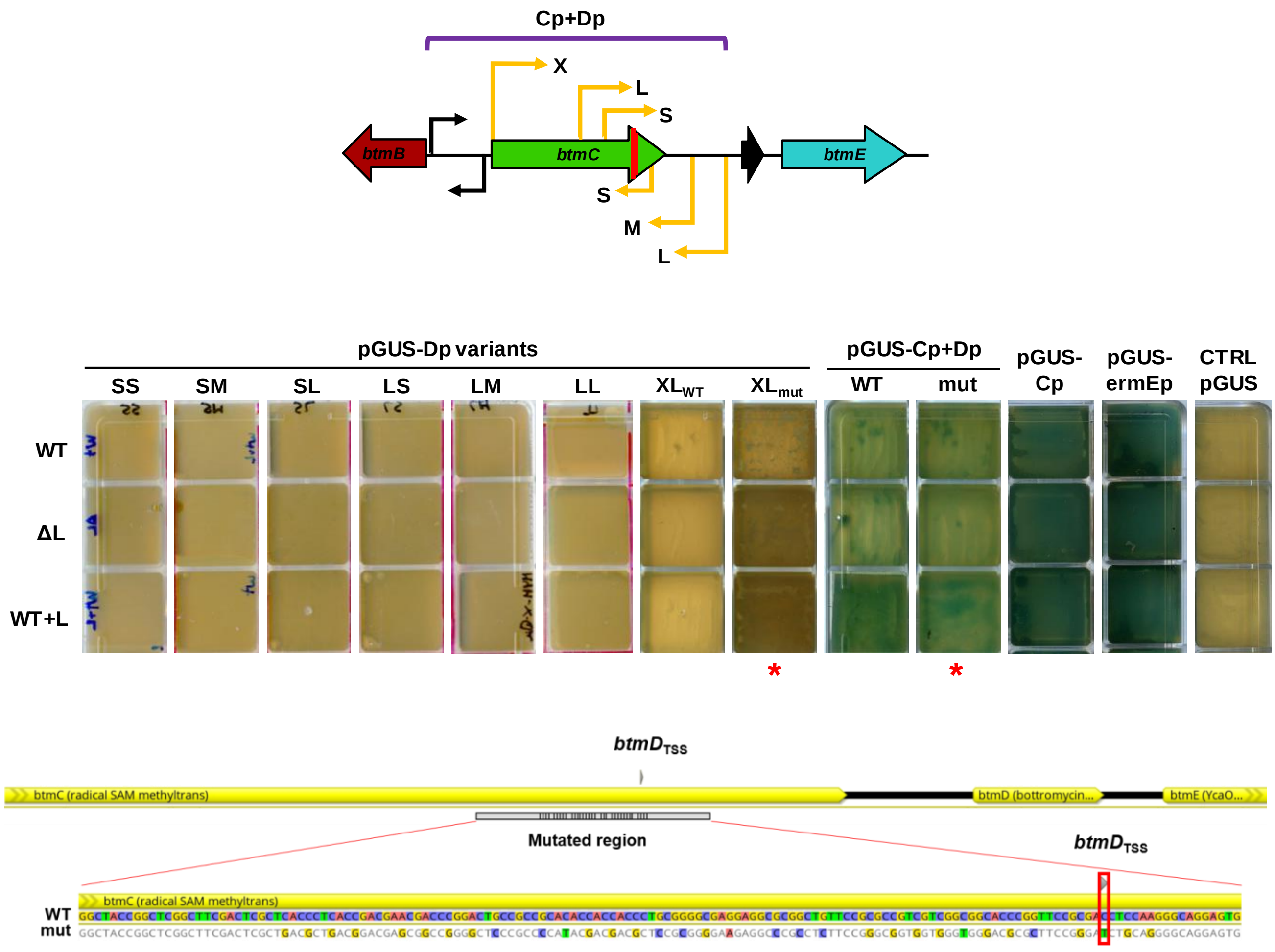


**Supplementary Figure S4.** Promoter activity of *btmD*p variants using β-glucuronidase (GUS) reporter assays in solid medium. A set of primers annealing in the *btmD* promoter region (top diagram) were used in different combinations in order to generate different length probes for transcriptional fusion with *gusA*. These fusions were tested in qualitative GUS assays in solid medium. Each of the squares represents one independent culture, with squares in the same row corresponding to cultures carried out in the same genetic background and each column corresponding to a specific putative promoter region tested. The vectors containing *btmC*p (pGUS-Cp) *ermE**p (pGUS-ermEp) and promoterless pIJ10742 (CTRL pGUS) were used as controls in this experiment. For the longest transcriptional fusions, pGUS-Dp_XL and pGUS-Cp+Dp, both the WT sequence and a mutant version (“mut” fusions, marked with a red asterisk in the GUS experiment) were tested. The bottom diagram shows an aligment of the WT and mut sequences, highlighting the 33 point mutations (including one on *btmD*$_{TSS}$) present in the mutant version of these promoter fusions.

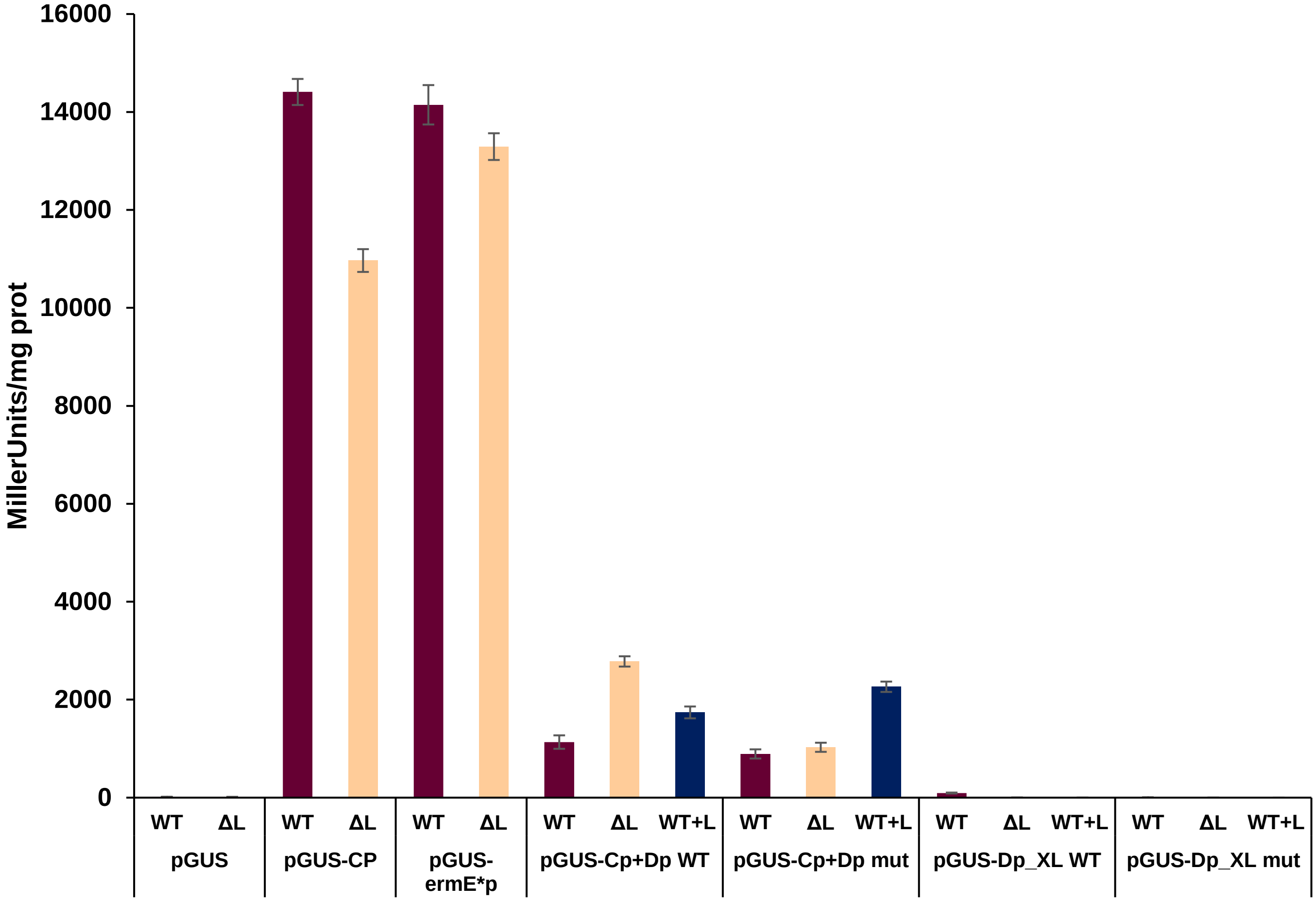


**Supplementary Figure S5.** Quantitative analysis of promoter activity of *btmD*p variants using β-glucuronidase (GUS) reporter assays in liquid medium. The longest transcriptional fusions tested in the previous experiment, pGUS-Dp_XL and pGUS-Cp+Dp, both in their WT and a mutant versions were used for quantification of their promoter activities in liquid bottromycin production medium (BPM). The vectors containing *btmC*p (pGUS-Cp) *ermE**p (pGUS-ermE*p) and promoterless pIJ10742 (pGUS) grown in S.scabies WT and *ΔbtmL* were used as controls in this experiment. Reporter activity expressed as Miller units/mg protein.

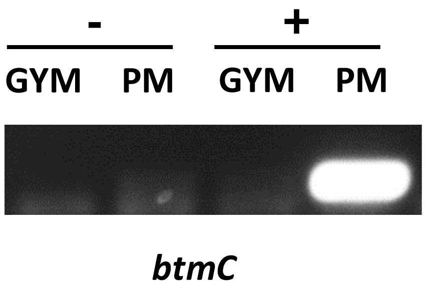


**Supplementary Figure S6.** RT-PCR analysis of the cultures used for the DNA affinitiy capture assay. Expression of *btmC* was assessed in 72h cultures of *S. scabies* both in GYM and BPM. Lanes marked with “-“ correspond to negative controls to discard DNA contamination.

**Supplementary Table S3.** Filtered list of proteins captured in the DNA affinity pull-down experiment that bind to *btmC*p in bottromycin production conditions. *Ratio of the normalized weigh spectra counts of the protein hit in BPM (production condition) over its value in GYM (non-production condition). **Annotation of the protein in the proteomics database. ***Prediction of domains or potential function of the protein based on BLAST and Pfam searches, and Phyre2 *in silico* modelling.

| Identified Protein | MW (kDa) | Fold Change* | Fisher's Exact Test (p < 0.02957) | Annotation** | Prediction*** |
|---|---|---|---|---|---|
| **SCAB_55281** | 25 | INF | 0.001 | Putative two-component system response regulator | MtrA |
| **SCAB_61751** | 12 | INF | < 0.00010 | Nitrogen regulatory protein P-II (GlnB) | GlnK |
| **SCAB_85931** | 32 | INF | 0.0032 | Putative regulatory protein | RsbR-like anti-anti-sigma factor |
| **SCAB_49851** | 22 | 4.7 | 0.00013 | Uncharacterized protein | Putative lipoprotein |
| **SCAB_51451** | 49 | 31 | < 0.00010 | Uncharacterized protein | Putative transcriptional regulator |
| **SCAB_33361** | 70 | INF | < 0.00010 | Uncharacterized protein | Histidine Kinase-like ATPase, putative DNA mismatch repair protein |
| **SCAB_29811** | 33 | INF | < 0.00010 | Uncharacterized protein | DUF4098 containing protein |
| **SCAB_30961** | 28 | INF | < 0.00010 | Uncharacterized protein | Ferritin-like diiron containing prot, putative tRNA hydroxylase |
| **SCAB_84851** | 30 | INF | 0.0004 | Uncharacterized protein | S-adenosyl methyltransferase |
| **SCAB_56661** | 52 | INF | 0.0004 | Uncharacterized protein | BtmF |
| **SCAB_30591** | 50 | INF | 0.0004 | Uncharacterized protein | DUF2342, putative hydrolase |
| **SCAB_34071** | 28 | INF | 0.0022 | Uncharacterized protein | von Willebrand factor A domain-containing protein |
| **SCAB_1641** | 34 | INF | 0.0038 | Uncharacterized protein | Ferritin-like diiron containing prot, AurF-like |
| **SCAB_33291** | 91 | INF | 0.0022 | Uncharacterized protein | DUF262 (unknown), DUF1524 (putative endonuclease) |
| **SCAB_41771** | 24 | INF | 0.0007 | Uncharacterized protein | DUF5063 containing protein |
| **SCAB_12301** | 49 | INF | 0.02 | Uncharacterized protein | TIR and FxsC domain containing protein |
| **SCAB_32431** | 112 | INF | 0.0066 | Uncharacterized protein | CHAT domain containing protein |
| **SCAB_17531** | 89 | INF | 0.012 | Uncharacterized protein | PAS and phosphatase domain containing protein |
| **SCAB_88431** | 16 | INF | 0.012 | Uncharacterized protein | Unknown |
| **SCAB_25431** | 19 | INF | 0.02 | Uncharacterized protein | N-acetyltransferase |
| **SCAB_28591** | 14 | INF | 0.012 | Uncharacterized protein | Unknown |
| **SCAB_5811** | 13 | INF | 0.012 | Uncharacterized protein | Unknown |

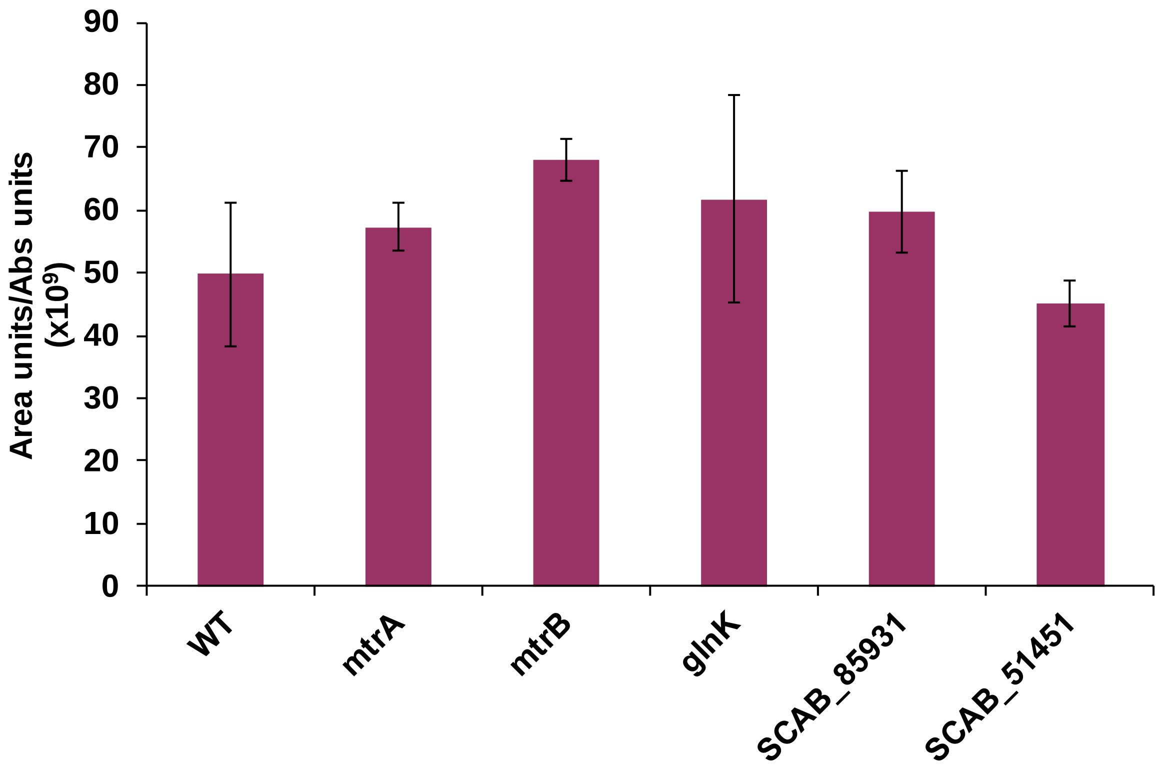


**Supplementary Figure S7.** Effect of potential transcriptional activators of the bottromycin pathway on antibiotic production. Each of the selected genes were overexpressed in *S. scabies* WT and the bottromycin production of the resulting strains plus the WT was analysed by LC-MS. Error bars represent the standard deviation of biological triplicate data and the production values are normalized by culture growth.
